# Specific social challenges for rare diseases: the French experience, 2005-2014

**DOI:** 10.1186/1750-1172-9-S1-O30

**Published:** 2014-11-11

**Authors:** Christel Nourissier

**Affiliations:** 1 Alliance Maladies Rares, EURORDIS

## 

The first strategic Plan in the world with a comprehensive approach for rare diseases was implemented in France from 2005 to 2008, followed by a second Plan in 2011 [1]. The 1st Plan had one objective: ensuring equity in the access to diagnosis, treatment and provision of care. The Second Plan aims at improving patient’s and carers’ health and social care, as well as developing research and European and international cooperation.

Among the major achievements of the 1st Plan was the designation and support of 131 National centres of expertise in University hospitals, coordinating networks of 500 centres of competence at regional level. One of the missions of those centres is the coordination with primary care, medical and social care. In the framework of the 1st Plan, pilot networks were created by two centres together with patient organisations and local professionals, and funded by the Regional Health Agencies of Pays de la Loire and Languedoc-Roussillon.

It is now understood that the interface of centres of expertise with social services is essential, when taking care of severely disabling and rare diseases, to facilitate clinical research and clinical trials, prevent the burn-out of medical teams and the explosion of medical costs. Highly specialised hospital care is inefficient if patients do not access adapted schooling, employment, housing and social services at home. Without adapted support, they soon return to the hospital. Moreover, specialised social care is much cheaper than hospital care, and people have a better quality of life.

Today the regrouping of existing centres of expertise, diagnosis and research laboratories, patient associations, social professionals and care networks is encouraged to share resources and tools and cover all rare diseases and patients with unclear diagnosis in the long term. Following a call for proposals in 2013, 20 to 25 disease networks are being identified and supported. Disease networks and clusters of centres of reference at regional level are encouraged to establish links with the local authorities in charge of the compensation of disabilities « Maisons Départementales des Personnes Handicapées (MDPH) », participate in workshops to improve the use of medical certificates, develop a common language and common evaluation tools. Following the creation of international codes for rare diseases, Orphanet is now assessing consequences of rare diseases on disabilities in daily life using WHO ICF-CY 2007, and developing disease specific fact sheets [2].

All rare diseases are not associated to rare disabilities [3]. However 65% of rare diseases are associated to multiple disabilities. People with complex dependency needs do not find adequate support in existing social services: they require pluridisciplinary evaluations, collaboration of different local services, existing centres of expertise and resource centres, and complex cases managers if available. Coordination and good will are not enough. Integration of all available health and social services is needed to support inclusive and continuous life trajectories.
